# Architecture design and advanced manufacturing of heart-on-a-chip: scaffolds, stimulation and sensors

**DOI:** 10.1038/s41378-024-00692-7

**Published:** 2024-07-11

**Authors:** Feng Xu, Hang Jin, Lingling Liu, Yuanyuan Yang, Jianzheng Cen, Yaobin Wu, Songyue Chen, Daoheng Sun

**Affiliations:** 1 Pen-Tung Sah Institute of Micro-Nano Science and Technology, Xiamen University, Xiamen, 361102 China; 2 Guangdong Provincial People’s Hospital, Guangzhou, 510080 China; 3 School of Basic Medical Sciences, Southern Medical University, Guangzhou, 510515 China

**Keywords:** Microfluidics, Electrical and electronic engineering, Biosensors

## Abstract

Heart-on-a-chip (HoC) has emerged as a highly efficient, cost-effective device for the development of engineered cardiac tissue, facilitating high-throughput testing in drug development and clinical treatment. HoC is primarily used to create a biomimetic microphysiological environment conducive to fostering the maturation of cardiac tissue and to gather information regarding the real-time condition of cardiac tissue. The development of architectural design and advanced manufacturing for these “3S” components, scaffolds, stimulation, and sensors is essential for improving the maturity of cardiac tissue cultivated on-chip, as well as the precision and accuracy of tissue states. In this review, the typical structures and manufacturing technologies of the “3S” components are summarized. The design and manufacturing suggestions for each component are proposed. Furthermore, key challenges and future perspectives of HoC platforms with integrated “3S” components are discussed.

**Figure:**
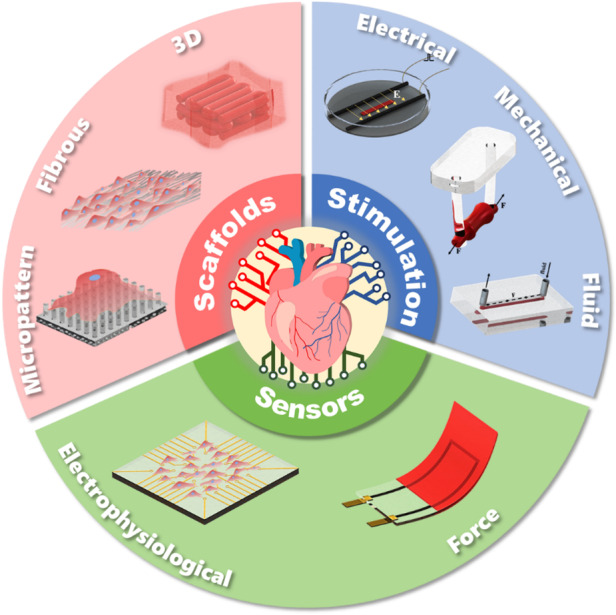
Architecture design concepts of scaffolds, stimulation and sensors in chips.

Architecture design concepts of scaffolds, stimulation and sensors in chips.

## Introduction

Heart diseases, such as myocardial infarction, hypertrophy and atherosclerosis, are the most common causes of death worldwide, increasing the demand for novel cardiac drugs. However, due to adverse reactions in the clinical stage or cardiotoxicity, most drugs do not undergo premarket assessments or must be withdrawn from the market due to health risks. Engineered cardiac tissue may provide an in vitro model and alternative to preclinical animal models with fewer ethical issues and lower costs^1,2^. A delicately designed heart-on-a-chip (HoC) is expected to produce highly mature engineered cardiac tissue that resembles native tissue in morphology, gene expression, and electromechanical properties^3–5^.

Cardiac tissue contracts rhythmically in response to electrical signals generated from the sinus node. This routine task involves close cooperation among the whole heart, which is characterized by an anisotropic cardiac tissue structure with a global stiffness (5–30 kPa); electrophysiological properties, including a conduction velocity of ~15 cm/s; and a mechanical beat with a contractive force of ~50 mN/mm^2^ and a strain of ~25%^6–8^. Among the factors that influence tissue maturation in HoCs, the multilevel microstructures of the extracellular matrix, bioelectricity, and contractility play crucial roles in material-energy-information transfer in cardiac tissue.

**Fig. 1:**
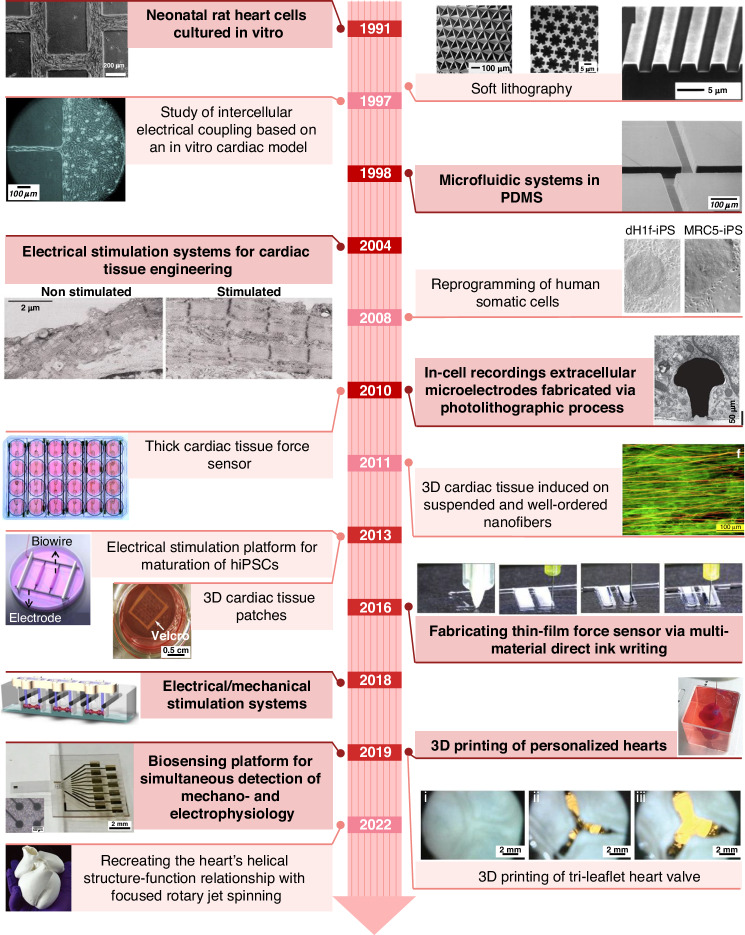
Major milestones in cardiac tissue scaffolds, stimulation, and sensors architectures and applications^9–21,75,225–229^

In studies on engineered cardiac tissue, the following key components were commonly mentioned: cardiac tissue scaffolds provided support and induced tissue growth, electrical and mechanical stimulation promoted tissue maturation, and various sensors detected the status of tissues. By constructing scaffolds, stimulation, and sensors on chips, researchers can reproduce and monitor the cardiac tissue culture mechanical/electrical microenvironment. However, the architecture design and manufacturing of HoCs must satisfy high requirements to achieve this integration. Due to the emergence of new materials and advances in manufacturing technologies, multimaterial, functional and delicate structures have been formed. The development of scaffolds, stimulation, sensors, and their integration on chip has accelerated the maturation of engineered cardiac tissue and methods to monitor in situ dynamic behavior, extending the functions of HoCs and their application in clinical trials. Representative studies on advancements in tissue engineering and advanced manufacturing applied to HoCs have been chronologically summarized, and a historical timeline of major events is presented in Fig. 1.

Scaffolds, as the central part of tissue culture and supporting tissue growth, have been a hot topic in tissue engineering research since 1991. Morphologically, scaffolds have developed from 2D inert substrate and planar microstructure scaffolds to multilayered fibrous structures and complex 3D scaffolds^9–14^. To further promote the maturation of engineered cardiac tissue, on-chip stimulation has been proposed as an effective tool. In 2004, electrical stimulation was shown to greatly improve the physiological properties of cardiac tissue. In 2018, simultaneous electrical/mechanical stimulation systems were built to induce adult gene expression and form remarkably organized ultrastructures^9,15–18^. In 2022, focused rotary jet spinning (FRJS), an innovative additive manufacturing method, was developed to construct micro/nanofiber scaffolds with helically aligned 3D geometries^19^. High-throughput, on-chip integration of flexible electrical/force sensors is also essential for improving efficiency during tissue culturing or drug testing. This integration of fully functional HoCs extended their applications in drug screening or clinical trials^18,20,21^.

In this review, we summarize the current architecture design of “3S” components, scaffolds, stimulation, and sensors, as well as their corresponding advanced manufacturing technologies. The mechanisms underlying the interplay between individual components and cardiomyocytes (CMs) have been elucidated, but this growing body of research remains in its early stages. Therefore, we provide insights into cardiomyocyte maturation in the field of engineered cardiac tissue. Although biochemical modification and stimulation are very important techniques^22–24^, they are not considered in this review. We are dedicated to providing researchers with profound knowledge on the architectural design of functional components, manufacturing technologies, and their ability to modulate cardiac tissue properties, and we believe that the emergence of new materials and manufacturing technologies will further promote the development and application of HoCs.

## Scaffolds

Scaffolds act as the core component of HoCs, providing support for the cardiac tissue. After cardiomyocytes are seeded into the scaffold, they undergo the following processes: adhesion, induced growth, and cultivating maturity. Cardiac cells exhibit adherent growth, and dissociated and suspended free cells cannot survive. Therefore, the first key step in cell culture is to provide adherent substrates^25^. Through adhesion, cells can sense the mechanical properties of the matrix and eventually shape the tissue^26^. Fibrillar collagens in the ECM constrain and induce cardiomyocyte growth through focal adhesions, while the cytoskeleton can detect the direction of mechanical load and then dynamically adapt to the mechanical load imposed by matrix stiffness^27^. The scaffold stiffness information and external mechanical forces are transmitted through the myofibril skeleton/focal adhesion/scaffold^28^. A scaffold that structurally resembles natural ECM can promote cell maturation and enhances gene and protein expression^29–34^.

When designing cardiac tissue scaffolds, it is crucial to a. choose materials with suitable stiffness or design microstructures with appropriate stiffness to enhance scaffold-cell adhesion; b. apply biochemical treatment to the scaffold surface to foster focal adhesions; c. design anisotropic geometries to induce cardiac tissue orientation; and d. utilize suitable porous 3D structures to simulate natural tissue environments. In this section, 2D and 3D scaffolds are discussed, with the main focus on their structural design, manufacturing, mechanical properties, and effects on tissue maturity.

### Two-dimensional scaffolds

**Fig. 2:**
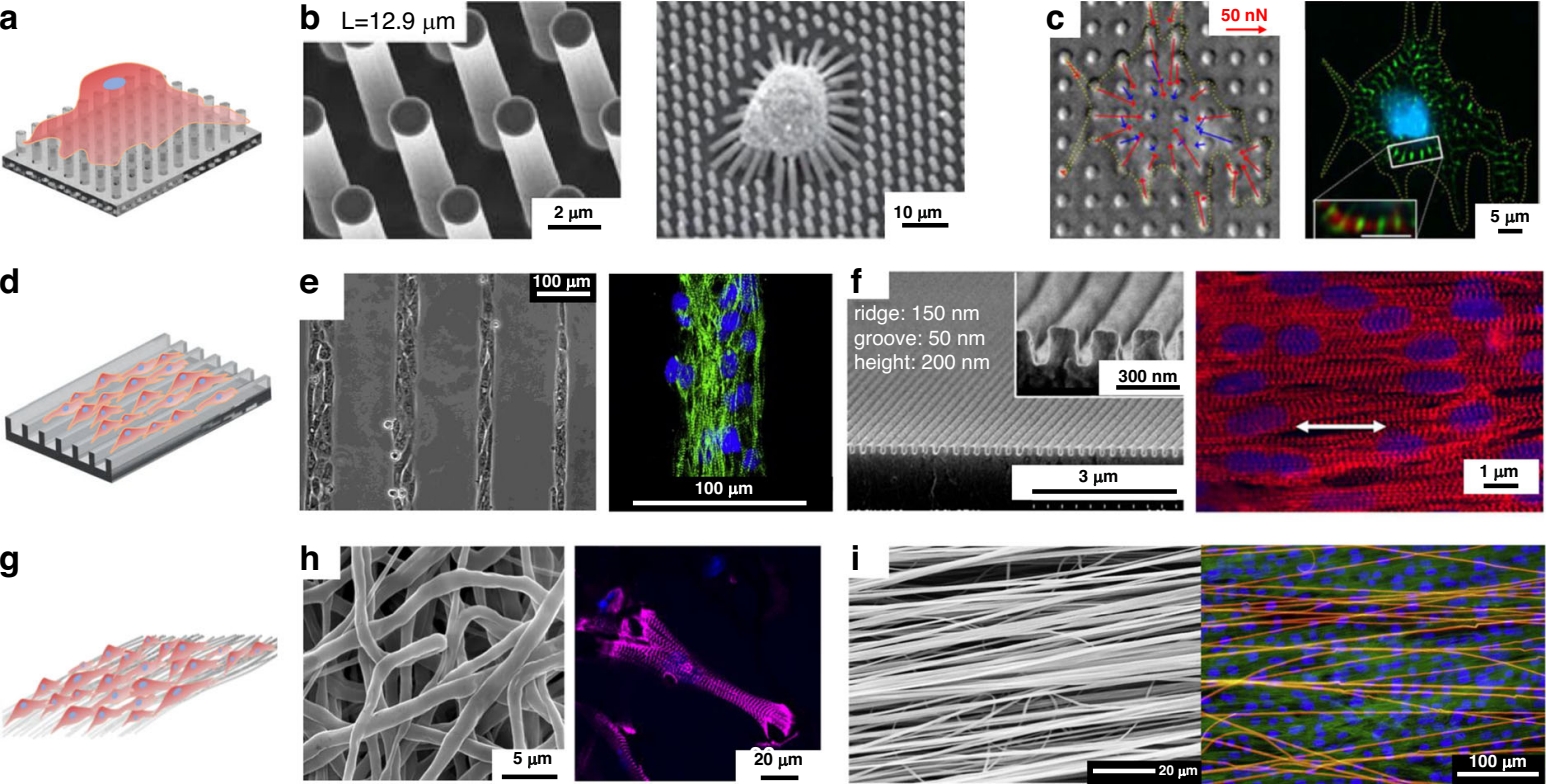
2D scaffolds. Point contact constraint structures: **a** Schematic diagram of point contact constraint; **b** PDMS micropillar arrays^14^; **c** PDMS micropillar arrays coated with fibronectin^36^; Some groove structures: **d** Schematic diagram of groove constraint; **e** Silicon substrates containing micron-sized notches^43^; **f** PEG nanogrooves^46^; Fibers constraint structures: **g** Schematic diagram of fiber constrained structure; **h** Disordered PCL fibrous networks^51^; **i** Ordered nanofiber^10^

Culturing cardiomyocytes on a 2D plane is the most widely used approach in tissue engineering. The stiffness scaffolds are generally regulated by using flexible materials or designing micro/nanostructures with low stiffness. The structural and stiffness of the scaffolds determine the distribution of focal adhesions and the formation of signaling pathways. The physical arrangement of focal adhesions plays a pivotal role in cellular differentiation and maturation^35^, while the introduction of micro/nanomorphology, including micropillar arrays (Fig. 2a), micro/nanogrooves (Fig. 2d), and fibrous networks (Fig. 2g), enhanced the functionality and diversity of scaffolds.

#### Micropillar array scaffolds

Flexible micropillars were fabricated as 2D scaffolds to study the effect of substrate stiffness on cell performance. The stiffness of the micropillars was adjusted through size control. Polydimethylsiloxane (PDMS) micropillar arrays were prepared by demolding with silicon molds, which were manufactured by photolithography and deep reactive ion etching (Fig. 2b)^14^. Fu et al. fabricated micropillars with a diameter of 1.83 μm and height of 0.97 to 14.7 μm, which led to an in rigidity from 1.31 nN/μm (for *h* = 14.7 μm) to 1556 nN/μm (for *h* = 0.97 μm), which is more than 1000-fold. In vitro assays have shown that the stiffness of the scaffold plays a role in regulating the twitch forces produced by immature cardiomyocytes. Rodriguez et al. inoculated neonatal rat cardiomyocytes (NRVMs) on PDMS micropillar arrays coated with fibronectin at different stiffnesses (Fig. 2c) and concluded that NRVMs cultured on stiff scaffolds (20 kPa) had greater calcium activity and contractive force than those cultured on soft scaffolds (3 kPa)^36,37^. The isotropic micropillar array provided a platform for single-cell analysis; however, the platform could not provide an anisotropic culture for cardiac tissue^38–42^.

#### Micro/nanogroove scaffolds

A common type of 2D geometric pattern constraint is micro/nanogrooves. An orderly cell arrangement results in a higher unidirectional force output. Fluted structures provide constraints through their wall-bottom-ridge surface structure^43^. Micro/nanogrooves of different widths and heights were prepared by soft lithography or other nanofabrication techniques to improve the contact induction effect of the scaffolds on cardiomyocytes. Motlagh et al. fabricated silicon substrates containing micron-sized notches (with groove and ridge widths of 5–10 μm and groove depths of 2–5 μm) via photolithography (Fig. 2e)^44^. Cardiomyocytes were significantly more aligned (46.9 ± 4.3%) compared to their flat counterparts (2.9 ± 0.95%), which rose to 69.8 ± 2.0% on the grooves with a depth of 5 μm. Rao et al. fabricated microgrooves on PDMS substrates with a groove width of 10 μm, a gap of 10 μm, and a depth of 4 μm^45^. Induced pluripotent stem cell-derived cardiomyocytes (iPSC-CMs) cultured on these substrates exhibited more organized sarcomeres, and the Ca^2+^ cycling properties were significantly improved in terms of speed and amplitude. Multiple nanoscale grooves in contact with a single cardiomyocyte provide more uniform and dense confinement. Kim et al. used UV-assisted capillary lithography-based nanomolding techniques to fabricate PEG nanogrooves (Fig. 2f). Compared with CMs cultured on a flat substrate, CMs cultured on nanogrooves were 1.6 times longer^46,47^. However, groove depths below 35 nm or ridge widths below 100 nm did not induce fibroblast alignment, and the cells were no longer guided along the nanogroove pattern^48^. This result suggested that the resolution of topographic sensing by cells was at the submicron level^49^.

#### Fibrous scaffolds

Due to their tunable physicochemical properties, electrospun nanofibers have been explored as a tool to control architecture in cardiovascular tissue engineering^50^. Cardiomyocytes can be confined by controlling the manufacturing process to regulate the diameter, orderliness, and surface biochemical properties of the fibers and induce tissue growth^51^. Fleischer et al. prepared random PCL fibrous networks with three different diameters (300 ± 100 nm, 1.3 ± 0.07 μm, and 2.8 ± 0.13 μm) (Fig. 2h)^51^. The researchers discovered that cardiomyocytes on micron-scale fibers possessed a greater aspect ratio than those on nanoscale fiber scaffolds. Similarly, cardiomyocytes with a greater aspect ratio were found on 3.6 μm diameter fibers than on 0.14 μm and 0.76 μm fibers^52^. Within a certain diameter range, micron-sized fibers provided stronger line constraints to the cells, causing the cells to stretch in the direction of the fibers; therefore, a morphology with a higher aspect ratio could be obtained.

The orderly arrangement of cardiomyocytes is another sign of tissue maturation. In the early days, oriented textured scaffolds were prepared by mechanical stretching, which places high demands on the elasticity of the scaffold materials^53^. Highly aligned polypropylene-co-glycolide (PLGA) nanofibrous scaffolds (~50 μm) were prepared using roller electrospinning, in which cultured human-induced pluripotent stem cell-derived cardiomyocytes (hiPSC-CMs) grew in a fibrous orientation with a longer sarcomere length (~1.68 μm), faster Ca^2+^ propagation rate, and more mature electrophysiological properties than those on petri dishes^54^. In addition to orientation, fiber density is an important issue in obtaining an oriented arrangement and ensuring that cell communication occurs perpendicular to fibers. Orlova et al. used aluminum foil with rectangular pores to induce electrospinning of aligned fibrous networks at different densities (Fig. 2i)^10^. It was found that a single fiber carried one or a few cells at a density of 5–10 fibers/mm without cell communication; at 20 fibers/mm, interlaced nanofibers could form a few streaks of tissue; and at a density higher than 50 fibers/mm, aligned fibers could induce more pronounced cell alignment and directional growth of tissue.

### Three-dimensional (3D) scaffolds

3D scaffolds provide cells with a 3D spatial growth environment, establishing physiological cell-cell and cell-extracellular matrix interactions in vitro. 3D scaffolds were initially fabricated by a layer-by-layer stacking process with 2D scaffolds. However, the simple stacking process limited the spatial distribution and porosity of the scaffolds, leading to difficulties in cell perfusion and tissue survival. Porous 3D scaffolds, which mimic the native ECM structure, improved the seeding and cultivation performance of thick cardiac tissue by reducing the diffusion pathways for cell migration, culture solutions and oxygen supply^35^. With advancements in hydrogel materials and 3D printing technology, feasible solutions involving the integrated fabrication of multifunctional porous and volumetric scaffolds have been proposed.

#### Porous scaffolds

In native cardiac tissue, the 3D porous structure of the ECM determines the spatial distribution of focal adhesions, allowing for 3D mechanical signaling between cells and the matrix^55–57^. This 3D binding force is transmitted into the cell as an external mechanical signal via focal adhesion, which guides the orientation of myogenic fibers and translates into biochemical signals; these signals then alter protein synthesis and gene transcription and promote electrical coupling among cells^58,59^.

Hydrogel consists of hydrophilic polymers crosslinked by covalent bonds or physical attraction to form a 3D network; through this network, the hydrogel can absorb large amounts of water (up to 99%). Hydrogels are soft and elastic and largely resemble the ECM^60^. Bryant et al. designed and fabricated porous, biodegradable poly(2-hydroxyethyl methacrylate) hydrogel scaffolds via photolithography^61^. Open, vertical channels ranging from 360 to 730 μm in size were patterned into scaffolds with pore diameters of 62 or 147 μm. However, a high degree of orientation could not be easily induced to the cardiac tissue using the random pore structure^62^. Bian et al. cast a C2C12 myogenic cell/hydrogel mixture in a culture area with staggered elongated PDMS columns^62,63^ to control the arrangement of cells in hydrogels with geometric constraints. The resultant tissue exhibited an interconnected, dense, and uniformly aligned morphology and contracted spontaneously. Compared to 2D monolayers, hESC-CMs in 3D hydrogel patches exhibited significantly greater conduction velocity and longer sarcomeres^11^.

**Fig. 3:**
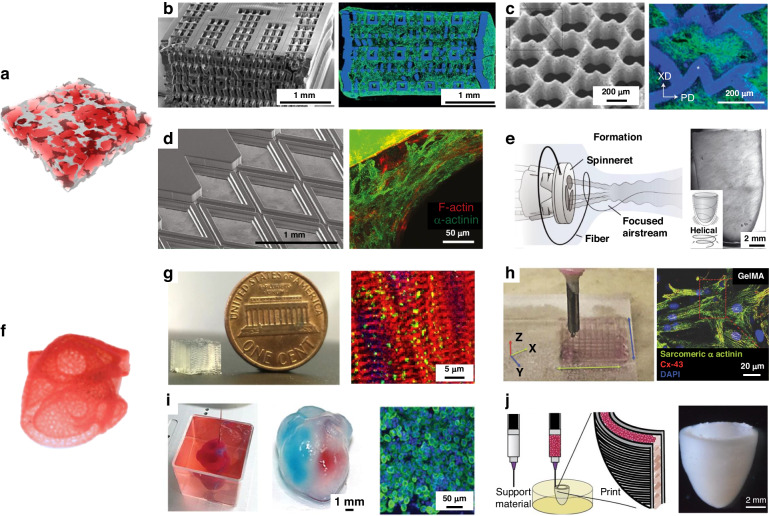
3D scaffolds. Typical porous scaffolds: **a** Schematic diagram of porous scaffolds; **b** Porous poly(octamethylene maleate (anhydride) citrate) (POMaC) scaffolds^64^; **c** Accordion-like honeycombs scaffolds^66^; **d** POMaC scaffolds with rhombic pores^230^; **e** Ventricles-like fibrous scaffolds^68^; 3D scaffolds with volumetric structure: **f** Schematic diagram of volumetric structure; **g** DIW 3D microfibrous hydrogel scaffolds^69^; **h** Gold Nanocomposite Bioink^70^; **i** 3D Printing of Personalized Thick and Perfusable Cardiac Patches and Hearts^12^; **j** 3D bioprinting of collagen to rebuild components of the human heart^13^

Highly porous 3D scaffolds can be prepared through the combination of hydrogel and UV-curing technology. Radisic et al. designed a UV-curable elastomeric polymer (octamethylene maleate (anhydride) citrate) (POMaC) for these applications^64^. The Young’s modulus of the POMaC polymer could be adjusted (from 53 to 1423 kPa) to fit the tissue culture by modulating the UV intensity and exposure time, the monomer ratio and the concentration of the porogenic agent (Fig. 3b). The surface morphology of the rhombic scaffold constrained the cardiomyocytes and induced an orderly arrangement of stress fibers in the cardiomyocytes along the prismatic edges (Fig. 3d). The elastic modulus of the cultured tissue was 69.3 kPa and 14.7 kPa in the *x* and *y* directions, respectively, with an anisotropy ratio of 4.7, which is similar to that of rat cardiac tissue (anisotropy ratio of 3.9)^65^. Laser processing was employed to create an accordion-like honeycomb microstructure (Fig. 3c) using poly(glycerol sebacate) with controllable stiffness and anisotropy. These porous scaffolds could overcome the principal structural–mechanical limitations of previous scaffolds, promoting the formation of grafts with aligned heart cells; in addition, the scaffolds exhibited mechanical properties more similar to those of native myocardium^66^.

The electrospinning technique can be used to produce fibrous scaffolds with well-defined porosity and has been employed to fabricate 3D porous fibers. At present, the primary approaches for fabricating thick fibrous scaffolds involve layer-by-layer assembly processes and one-step scaffold fabrication. Wu et al. interwoved nanofiber mats layer-by-layer to induce the growth of cardiac muscle^67^. These 3D porous scaffolds, which contained an aligned conductive nanofiber yarn network, induced cellular orientation and maturation and were demonstrated to be an effective method for engineering 3D cardiac anisotropy. Chang et al. developed focused rotary jet spinning, a one-step method for manufacturing thick fibrous scaffolds. This method enabled the deposition of polymeric materials in the form of long, thin fibers with a preferred orientation at the micrometer scale^68^. Rapid fabrication of micro/nanofiber scaffolds with programmable alignments in 3D geometries (Fig. 3e) was achieved. Seeding these scaffolds with cardiomyocytes enabled the production of tissue-engineered ventricles, in which helically aligned models displayed more uniform deformations, greater apical shortening, and increased ejection fractions compared with circumferential alignments.

#### Bioprinted volumetric structure

The use of scaffolds with volumetric structures for preparing thick cardiac tissue holds great promise in the production of cardiac tissue patches. Bioprinting, employing hydrogel inks with living cells, was effective for fabricating complex volumetric structures, surpassing the capabilities of traditional perfusion approaches. A commercial bioprinter with a bespoke coaxial nozzle was used to print a bioink that contained a dual cross-linked system of alginate and gelatin methacrylate (GelMA)^69^. The structural integrity of the bioprinted scaffold could be ensured by coaxially extruding alginate with CaCl_2_ solution for ionic crosslinking, followed by photocuring GelMA to achieve permanent chemical gelation (Fig. 3g). Cardiomyocytes cultured on the printed anisotropic 3D scaffolds, with a modulus of 5.2 ± 0.9 kPa, exhibited a pronounced orientation, high CX-43 expression, and sustained contraction for up to 28 days. They bioinks were formulated by mixing gold nanorods with GelMA hydrogels through ultrasonic treatment^70^. GelMA microfibers doped with gold nanorods reduced the resistance of scaffold surfaces and improved cell-to-cell electrical coupling (Fig. 3h). The CMs on the 3D scaffolds that contained gold nanorods exhibited a greater frequency of synchronous contraction than that of CMs on the original GelMA/alginate bioink-printed scaffolds.

Aside from the emerging needs, thick tissue with bioprinted volumetric structures involves poor nutrient diffusion, resulting in core necrosis of tissues. Therefore, vascularized volumetric structures were developed to provide nutritional support to mimic tissues in vivo. Lee et al. presented a method to print collagen by embedding suspended hydrogel in a freeform reversible manner to engineer components of the human heart at various scales, from capillaries to the full organ^13^. This printing process utilized the temperature properties of the gelatin material by converting the material from a support layer to a sacrificial layer, resulting in a network of blood vessels with suspended pipes. Based on this principle, a neonatal-sized collagen heart was printed (Fig. 3j). Using a similar process, thick and vascularized 3D scaffolds were printed that perfectly matched the immunological, biochemical and anatomical characteristics of patients^12^. A small human heart with major vasculature was printed (Fig. 3i), which possessed mechanical properties similar to those of decellularized rat hearts.

## Stimulation

To facilitate cardiac tissue maturation, stimulation components are often designed based on electrical and mechanical stimuli, which are applied to which cardiomyocytes in vivo. The heart begins to beat in the embryo, in which the initial physical environment is formed. At this stage, the growth, shaping, and morphogenesis of the heart are promoted through stimulation rather than through nourishment^71^. Cardiac tissue contraction is caused by electrical signals from the sinus node, which are transmitted to all parts of the heart via the internodal bundle, the AV node, the bundle of Hitchcock, the right and left bundle branches and the Purkinje fibers. To mimic this process, electrical stimulation is applied to cardiac tissue using an electric field signal fed through external electrodes. As electrical signals propagate, rhythmic contraction and relaxation occur^72,73^. This dynamic activity, characterized by active contraction and passive stretching forces, enhances the mechanical properties of the tissue.

When blood is within a cycle that involves ventricular filling, isovolumetric contraction, ejection and relaxation, nutrients diffuse to supply the growth of cells; in addition, different impacts and shear effects on the whole heart are produced by fluid-stimulated environments, promoting progressive maturation of the cardiac tissue^69,74^. Additionally, directly applying cytokines, various biochemical factors and the oxygen supply the culture medium environment typically affects cells. The application of these stimulants is regulated by controlling the composition and injection method, and these stimulants are typically administered externally to HoCs; hence, this topic will not be further discussed in this review.

Stimulation exerts different effects on cardiomyocytes, which promote the maturation of tissues in various aspects, including electrophysiology, mechanical properties, morphology and gene expression. Therefore, the integration of multiple stimulation systems in HoCs could restore the physical environment in vivo, foster tissue maturation, and create a better biomimetic heart model (Table 1).

**Table 1:** Typical stimulation parameters

Cell type	Electrical stimulation	Mechanical stimulation	Morphology	Gene/Protein expression	Other performance	Ref.
hiPSC-CMs	1 ms, 3–4 V/cm, 1–6 Hz		Organized sarcomeric banding with frequent myofibrils, aligned Z discs	The fetal cardiac gene program (NPPA, NPPB, and MYH6) are downregulated	Excitation threshold ~1.5 V/cm, Maximum capture rate ~5.2 Hz, Conduction velocity ~15 cm/s	^17^
hiPSC-CMs	2 × 1 ms duration, 50 mA, 1 Hz	0–0.32 mm/day (tissue diameter 8 mm)	Displayed structural improvements in cellular volume, linear alignment, and sarcomere length (2.19 ± 0.1 µm)	Genes that are specifically expressed in adult cardiomyocytes are upregulated overall	The highest rate of stretch increased force development by 5.1-fold compared to tissue with a fixed length	^19^
hiPSC-CMs	Increases 0.33 Hz per day from 2 Hz to 6 Hz	Mechanical loading	Robust T-tubules, orderly registers of sarcomeres (length 2.2 μm) with I-bands, A-bands, M lines, Z lines, desmosomes, intercalated discs	The expression of genes and proteins related to conduction, ultrastructure, and calcium handling is increased	The resting membrane potential of −70.0 ± 2.7 mV, and the conduction velocity (25.0 ± 0.9 cm s^−1^)	^75^
hiPSC-CMs	2 ms duration, 0.5 Hz, 500 mV	SU-8 cantilever	Enhanced α-sarcomere actinin length (~1.7 μm)	The levels of Cx-43, α-sarcomere actinin, and TnT increase	The contraction force of cardiomyocytes is up to three times that of conventional cantilevers	^87^
hESC-CMs		PDMS molds	Longer sarcomeres (2.09 ± 0.02 μm)		Conduction velocity 25.1 cm/s, active stresses 11.8 ± 4.5 mN/mm^2^	^11^
neonatal mouse cardiomyocytes		PDMS cantilever	For Day 30 samples, the average sarcomere length was 2.34 ± 0.03 μm		The tension of tissues was 14.5 mN/mm^2^ at 1 Hz stimulation	^101^
hiPSC-CMs		10% uniaxial strain, 1 Hz frequency, 5 days	Troponin I exhibits an elongated form	The expression of CX43 and N-cadherin increased	The human cardiac microtissues began a spontaneous beating on Day 2.5 ± 0.5	^117^
embryonic Chick Cardiomyocyte		10 mmHg, ∼13% stretch at a frequency of 2 Hz, 4 days	Alignment of actin cytoskeleton, bundle-like sarcomeric α-actinin expression		Higher pacing beat rate at lower threshold voltages	^113^
hiPSC-CMs		Uniaxial gradual stress (a maximum strain of 2.3%@1 Hz)	Significant improvements in the sarcomeric alignment and localization of actin and troponin	GATA4,MYL2,MYH7 and CAMK2B were upregulated in the stimulated samples		^116^

### Electrical stimulation

Cardiomyocytes are a class of electrically excitable cells. Electrical stimulation was applied to train tissues to achieve better electrical transmission and mechanical performances. The application of an electrical field triggers an action potential in cardiomyocytes through transient ion flux, which establishes and maintains functional gap junctions; in addition, intracellular signaling pathways occur that establish cellular excitation, contraction, and coupling events^9,41^. It has been demonstrated that culturing cardiomyocytes using electrical stimulation methods during periods of high cell plasticity leads to the formation and functional development of their ultrastructure^75,76^. The architecture of the electrodes varied considering the stimulation voltage and electric field uniformity and can be classified into rod electrode structures and 2D patterned electrode structures.

The following aspects should be considered when designing electrical stimulation: a. ensure the biocompatibility of the electrode material; b. maintain a uniform stimulation electric field; c. maintain the applied electrical pulse within the non-Faraday response voltage range; and d. orient the electric field parallel (as much as possible) to the intended direction of cardiac tissue growth.

#### Rod electrodes

**Fig. 4:**
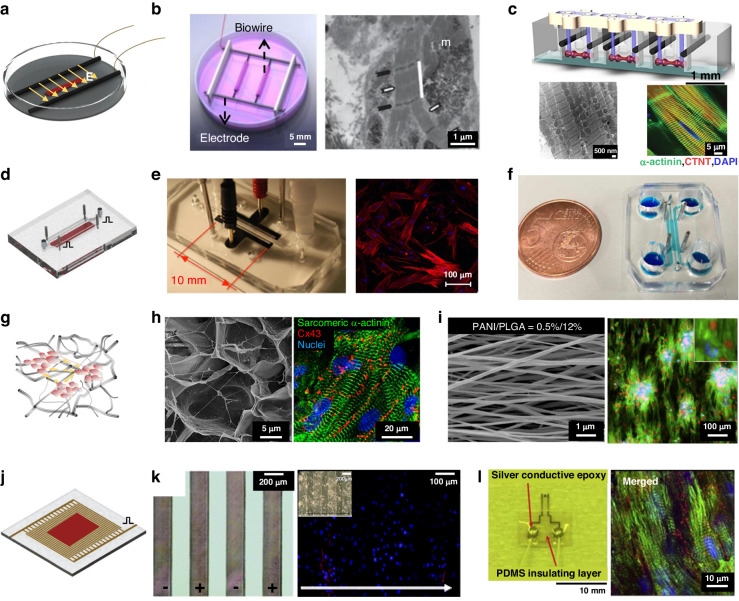
Typical structures for electrical stimulation. Show typical structures of rod electrodes: **a** Schematic diagram of rod electrodes; **b** Biowire with rod electrodes^17^; **c** Arrayed rod electrodes^75,76^; Show integrated rod electrodes structures: **d** Schematic diagram of integrated electrical stimulation on chip; **e** Integrated patterned counter electrodes on chip^80^; **f** Integrated stainless steel rods as electrical stimulation on chip^82^; Conductive scaffolds assist Rod electrodes structures: **g** Schematic diagram of conductive scaffolds; **h** Carbon-Nanotube-Embedded Hydrogel as conductive scaffolds^84^; **i** Conductive nanofibrous meshes^85^; 2D patterned electrodes: **j** Schematic diagram of patterned electrodes; **k** Surface-patterned electrode bioreactor^88^; **l** Micropatterned SU-8 cantilever integrated with metal electrode for enhanced electromechanical stimulation^87^

Due to their simplicity and reliability, rod electrodes have been used in most studies involving electrical stimulation. This method of stimulation delivers pulsed electrical fields to stimulate cardiac tissue through a pair of electrodes, which are placed on both sides of the culture environment^77,78^. Radisic et al. used a pair of 1/4 inch diameter carbon rods that were placed 1 cm apart, as shown in Fig. 4b^9^. The researchers applied rectangular pulses of 5 V/cm and 2 ms at 1 Hz to culture vessels and confirmed that electrical stimulation can directly affect the establishment and maintenance of functional gap junctions in NRVMs. In this study, NRVMs were cultured in vitro for 8 days, and the extent of tissue contraction increased 7-fold. When the ultrastructure was observed after electrical stimulation in culture, compact and visible M and Z lines were observed, as well as H, I, and A bands, which were comparable to those found in natural rat ventricular myocardium^17,41,75,76^. Zhao et al. developed the “Biowire2” platform, which uses a similar intensity of stimulation but a slower increase in frequency and a longer stimulation time^79^. The excitation threshold voltage (ET) and maximum capture rate (MCR) were monitored weekly, and the stimulation frequency was adjusted according to the MCR, resulting in a faster propagation velocity (31.8 ± 7.9 cm/s). Due to the indispensability of electrical stimulation in drug screening, on-chip electrical stimulation is designed that embed various stimulation electrodes on the chip, such as conductive polymer materials (Fig. 4e), stainless steel rods (Fig. 4f) and platinum wires^5,80–82^.

Different stimulation procedures have been proposed for culturing mature cardiac tissue with rod electrodes, which can be paired with a variety of two- and three-dimensional in vitro culture environments to provide a homogeneous electric field. However, the large size of the electrodes and electrode spacing and the required voltage pulse amplitude are usually above 3 V. The voltage increases the risk of electrode oxidation-reduction reactions and hydrolysis.

To improve electrical coupling between cells and further facilitate electrical stimulation, researchers designed conductive scaffolds. 3D scaffolds doped with conductive materials (carbon-based materials, Au nanomaterials, conductive polymers) were fabricated to create 3D conductive networks that alter the charge distribution at the scaffold-cell interface, thereby supporting cell adhesion, growth, attachment, and communication^83^. A conductive fibrous meshwork was formed by dispersing carbon nanotubes in GelMA hydrogels (Fig. 4h) with an impedance of ~5 kΩ at low frequencies^84^. The conductive CNT network improved intercellular Ca^2+^ transients and action potential propagation, providing an additional pathway for DC currents and reducing the impedance between cells. Therefore, a much lower threshold of electrical stimulation amplitude was needed, with an excitation threshold for ET reduction of 85%. Cardiac tissue cultured on CNT-GelMA showed several-fold improvements in marker protein expression and cell alignment, as well as a more even distribution of Cx43-linked proteins. Hsiao et al. transformed PANI/PLGA fibers into conductive scaffolds by doping with hydrochloric acid^85^. The introduction of a positive charge on the surface attracted negatively charged proteins, directing cell adhesion and orderly alignment (Fig. 4i). Isolated clusters of cells were coupled by electrical stimulation and contracted synchronously under stimulation with two silver electrodes.

#### 2D patterned electrodes

A gradient distribution of local pH at the electrode surface was induced by a high potential, and the engineered heart tissue was damaged by the production of gas. To achieve low-voltage stimulation (below a hydrolysis voltage of 1.23 V), planar microelectrode arrays were designed to reduce the electrode spacing while maintaining the stimulation electric field.

Planar electrodes can be combined with other stimuli and sensors to broaden the options of electrode materials and layout, providing excellent biocompatibility and flexibility for stable, well-repeated electrical stimulation. An Au thin-film electrode stimulation system (P-MEA) was fabricated and demonstrated good charge transfer characteristics in the range of 0.01 V to 1.0 V^86^. The cultured cardiomyocytes were arranged in an orderly fashion, with a contractile force of ~2 mN after stimulation. Oyunbaatar et al. deposited 100 nm thick gold counter electrodes on SU-8 cantilevers with a 1 mm electrode spacing (Fig. 4l) and stimulated hiPS-CMs for 7 days using a 0.5 V impulse voltage^87^. Due to the high amenability of patterning, the electrode array can be patterned to provide uniform voltage pulse stimulation at the bottom of the entire cell tissue. To favor optical observation, an array of ITO forked finger electrodes (with a gap of 200 μm) was micropatterned by an excimer laser (Fig. 4k)^88^. As the cardiac tissue was stimulated, ITO electrodes were used to capture real-time contractions of the tissue. Nevertheless, the electric field generated by 2D patterned electrodes was less uniform than that generated by rod electrodes, especially when thick tissues were stimulated^89^.

### Mechanical stimulation

Cardiac tissue actively contracts during periodic systole and diastole contractions. This continuous deformation exerts a locally directed cyclic uniaxial strain on the cardiomyocytes along the ECM. Mechanosensitive channels on cells are constantly stimulated by external strain, triggering diverse mechanotransduction signaling pathways, which ultimately promote cardiomyocyte maturation^90,91^. Mechanical stimulation was applied in vitro to replicate the in vivo conditions. Most of the structures demonstrated that cardiomyocytes subjected to suitable mechanical stimulation were morphologically more mature (larger, more facilitated cell alignment, longer sarcomere, Cx43 polarization) and showed a significant increase in ion channel protein expression and mechanical properties^92–97^.

The mechanical excitation that occurs when cardiac tissue is connected to an external device using a fixture has been extensively studied because the technology is simple to manufacture and achieves reliable performances for parametric experiments. Dendorfer et al. set up biomimetic cultivation chambers, which consisted of a membrane bonded to the ends of cardiac tissue in which one end was fixed and the other was connected to an actuator^19,98,99^. However, the maneuverability and stability of mechanical excitation based on fixtures are limited. Therefore, integrated mechanical excitation structures have been fabricated to provide passive or active stimulation to cardiac tissue, which eliminates the need for clamping and facilitates high-throughput excitation.

The following aspects should be considered when designing mechanical stimulation: a. a reasonable stimulation structure should be designed and uniaxial stretch should be applied to the tissue to induce tissue growth; b. the directional deformation of the mechanical stimulation structure should be considered, as cells from diverse sources display distinct tissue orientations poststimulation.

#### Passive stimulation structures

**Fig. 5:**
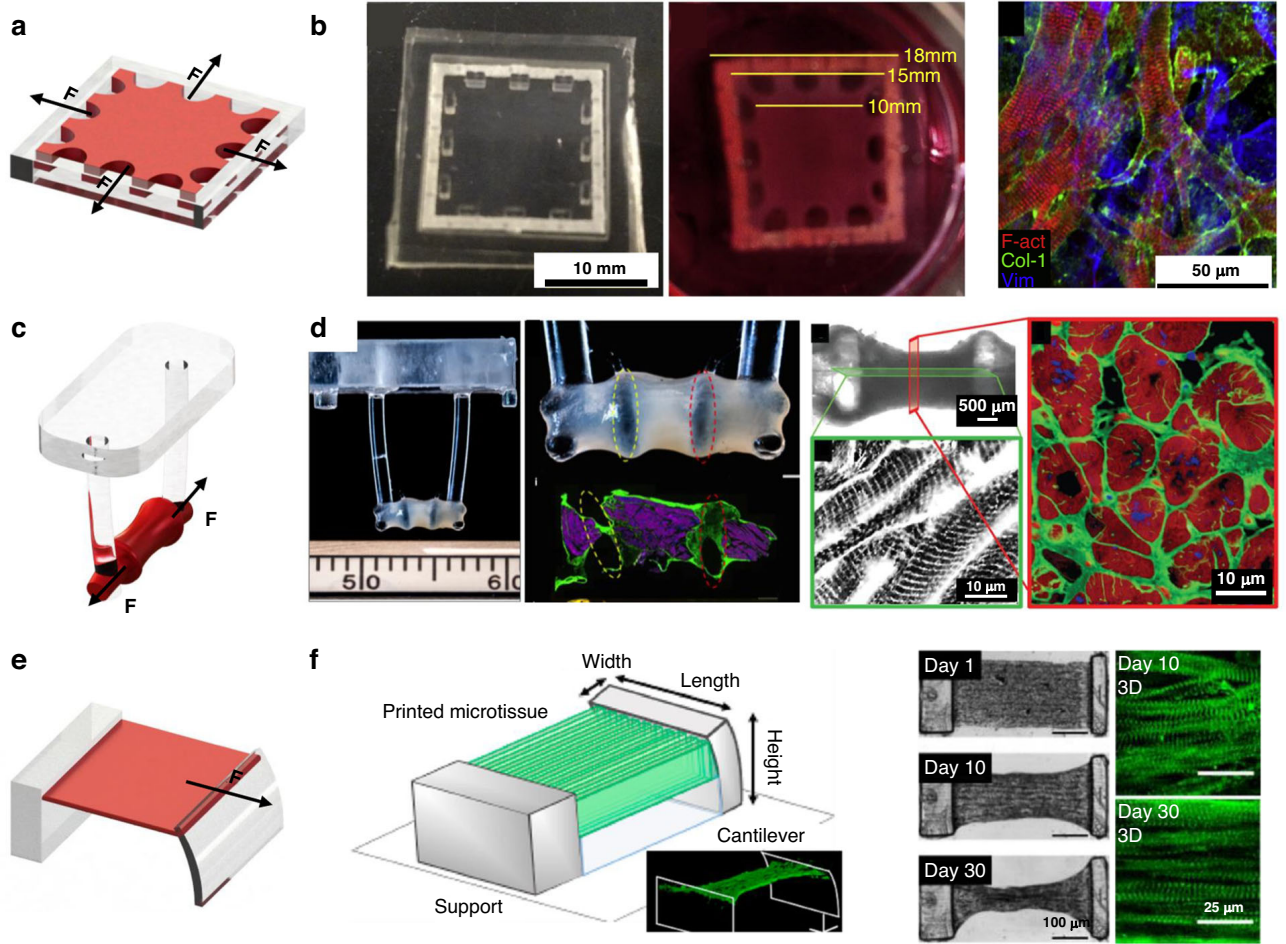
. **Typical structures for passive stimulation, a** Schematic diagram of flexible frame constrain; **b** Engineered cardiac tissue patch^100^; **c** Schematic diagram of flexible columns constrain; **d** Typical flexible column structures^75^; **e** Schematic diagram of cantilever beam; **f** Cantilever beam structures^101^

Passive mechanical stimulation can be achieved by the reaction force of a restriction frame (Fig. 5a,c,e). Nenad Bursac et al. manufactured human cardiac tissue patches with staggered elliptical pores^11^. A cell/gel solution was added to the PDMS mold and polymerized to create 3D tissue attached to an external flexible frame. When the tissue is pulsating, the flexible frame provides a counterforce. After two weeks of culture, 3D hESC-CM tissue exhibited increased contractility (3.0 mN), stress (11.8 mN/mm^2^) and sarcomere length (2.09 ± 0.02 μm) (Fig. 5b)^100^. Kacey et al. used flexible cantilever beam structures (Fig. 5d) to apply uniaxial restraint on tissue and generate ordered cardiac tissue with a sarcomere length of 2.2 μm via electrical stimulation training^75^. Liu et al. used microscale continuous optical printing to fabricate asymmetric, multimaterial cantilever beam structures (Fig. 5f)^101^. Simultaneously, hydrogels patterned with cardiomyocytes were crosslinked. The crosslinked 3D cardiac tissue hydrogels were constrained between the cantilever beams and started to beat synchronously after 3 days of incubation. Compared with the 2D samples, the 3D-encapsulated cardiac tissue generated almost twice the force. After 30 days of culture, the sarcomeres grew to ~2.34 μm and became more ordered.

When passive stimulation structures are used for mechanical stimulation, it is assumed that tissues can undergo spontaneous pulsation, which places high demands on the cardiomyocyte source and the culture effect.

#### Active stimulation structures

**Fig. 6:**
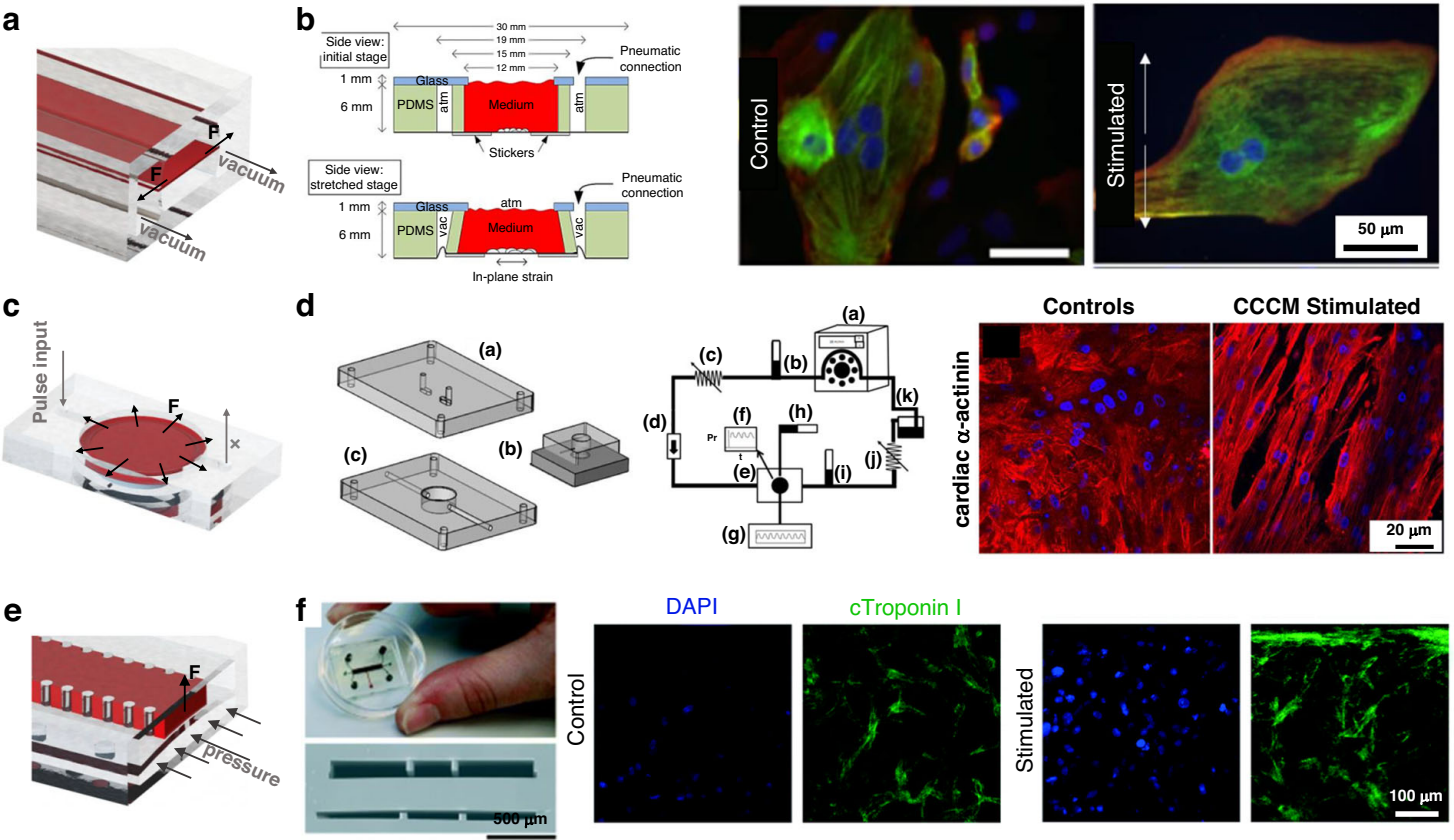
**Active mechanical stimulation structures**, **a**,**c** Two typical stretch structures; **b** Anisotropic stretch structure^107^; **d** Isotropic stretch structure^113^; **e** Schematic diagram of squeeze structure; **f** Integrated mechanical squeeze stimulation chip^117^

As the most widely used structure for on-chip active stimulation (Fig. 6a,c,e), the stretching structure causes the stretchable culture film to deform by contraction of the microchamber around the film. This structure allows the chip to adapt to almost any mechanical stimulation strategy, as the feature size can be adjusted^102–106^. Kreutzer et al. used this stretching structure (Fig. 6b) to apply a cyclic strain of 8% @0.8 Hz to cardiac tissue and observed that mechanical excitation increased the strength of the cardiac tissue and sarcomeres^107,108^. Notably, uniaxial stretching can be replaced with biaxial stretching, through which the effects of complex anisotropic forces on tissue maturation can be studied^109^. Pneumatic chambers were used to stretch the cardiac tissue, and a dielectric elastomer actuator (DEA) was placed symmetrically underneath the elastic membrane with an integrated MEA, with a high voltage to control the input for uniaxial stretching^110^. Compared to other methods, the mechanical stimulation applied via the DEA is more precise and facilitates the integration of electrical stimulation and sensors, allowing the minimization of the cardiac chip and its integration on a PCB board. Sethu et al. designed a microfluidic cardiac cell culture model (μCCCM), which can simulate the mechanical environment of the heart by applying different pressures, as shown in Fig. 6d^111–113^. The corresponding stimulated cells produced ~50% more RNA, increased total protein synthesis by ~37% and responded more significantly to isoproterenol. Embryonic chick cardiomyocytes are mechanically and electrophysiologically enhanced, and the corresponding RNA and protein expression is more mature due to mechanical stimulation^114^. However, biaxial gradual stress cannot induce cardiac tissue alignment^115^. In comparison, the application of uniaxial gradual stress induced better cell alignment and more active gene expression^116^.

Mechanical stimulation can also be induced through squeezing and stretching. Marsano et al. developed a HoC platform (Fig. 6f) that compresses CM gels to produce uniaxial strain, which could be stretched by applying pressure to the cavity below^117^. Cyclic pressure was applied to simulate the systolic and diastolic phases of the heart in a tunable manner. Stimulation of cardiomyocytes resulted in an elongated morphology of cardiac troponin I, increased Cx43 and N-calcine mucin density and spontaneous beating at 2.5 days.

## Mechanical and electrical sensors

Mechanical and electrical sensing on a chip is critical for promoting the use of HoCs as a high-throughput drug screening tool^118^ because chips with integrated scaffolds and stimulation provide a culture environment for tissue growth; more importantly, the integration of sensors allows real-time, in situ information recording of cardiac tissue. The mechanical and electrical characteristics of cardiac tissue, often regarded as the two pivotal indicators of its maturity, have been extensively studied. Therefore, force and electrophysiological sensors are two vital components of HoCs.

Optical observation is among the most widely used methodologies for assessing the characteristics of cardiac tissue and facilitates imaging for subsequent analysis and the extraction of mechanical and electrical signals^119,120^. For example, video analysis^121^ was employed to measure cardiac contractile force through calcium imaging^122^. However, indirect characterization of the mechanical and electrical performance cannot meet the needs of practical applications. Therefore, on-chip sensing was proposed, necessitating the implementation of delicate structural design and advanced manufacturing processes.

### Force sensor

The force sensors convert the contraction of tissue into a measurable deformation of a flexible structure. The amount of contractile force in cardiac tissue within hearts, a functional organ that pumps blood, is the most direct indicator of the maturity and physiological status of hearts. During Ca^2+^ influx, cardiomyocytes generate contractile force over the length of sarcomeres. By connecting the focal adhesion and adhesive bands, the force can propagate on the macroscopic scale, resulting in large-scale tissue deformation^22^. The contractile force of neonatal cardiac tissue is 0.8–1.7 mN/mm^2^, and that of mature tissue reaches >50 mN/mm^2^ ^123^. However, the active contraction range of hiPSC-CM tissue was ~0.05–23.2 mN/mm^2^ ^75,79,124,125^. Therefore, the force sensor must have the ability to measure microscale forces.

The following aspects should be considered when designing force sensors: a. a delicately flexible structural design that fits the limited space of HoCs should be implemented; b. the gradual process by which cardiac tissue matures should be considered and sensitive detection methods should be employed, which would allow the detection subtle of deformations caused by immature tissue.

Depending on the applicable scenario, force sensors can be divided into the following categories: single-cell sensors, thin-tissue force sensors, and thick-tissue force sensors. Microbead labeling, micropillar arrays, and Ca-transience are used to characterize the contractility of an individual cardiomyocyte by capturing the high-speed acquisition of changes in the position of markers through microscopy. Through atomic force microscopy, the beating force of cardiomyocytes can also be recorded by placing the cells in direct contact with nanoscale probes^37,120,121,126–141^. However, these methods for single-cell sensors require high-performance equipment and cannot meet the high-throughput requirements, hindering their large-scale application.

#### Thin tissue force sensing

Conventionally, the contraction force of thin cardiac tissue is converted and causes sensing structures to deform. Next, CellDrum and cantilever beams will be discussed.

**Fig. 7:**
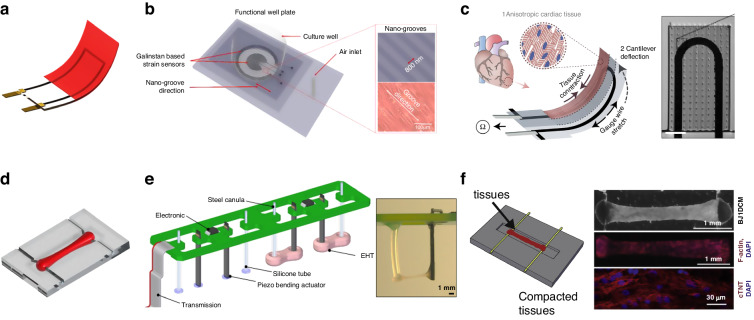
Force sensor structures. Show some typical sensor structures for thin tissue: **a** Schematic diagram of thin film force sensor; **b** CellDrum^144^; **c** Cantilever beam force sensor^20^; Sensor structures for thick tissue: **d** Schematic diagram of thick film force sensor; **e** Force sensing of flexible columns^161^; **f** A platform with flexible columns sensors^79^

CellDrum is a typical thin-film force sensing structure with a PDMS membrane and is fabricated by spin coating that seals the bottom of a cylindrical chamber. The contraction of the CM monolayer can be characterized indirectly by measuring membrane deformation. The key components of CellDrum technology are a well-defined, approximately isotropic, and homogeneous environment with biomechanical cell tension. Under pressure, the CellDrum membrane is deflected in the crown of a ball with radius R. For a spherical thin-walled pressure vessel of thickness, the tensile stress in the membrane can be calculated using Laplace’s formula as follows:

$$\sigma =\frac{p}{h}\frac{{r}^{2}}{4t}\left(1+\frac{{h}^{2}}{{r}^{2}}\right)$$

where p is the difference in air pressure between the inside and outside, h is the deflection distance and r is the radius of the CellDrum^142,143^. The contraction of the tissue can be calibrated according to the amplitude and frequency of the deflection from the flexible film, which can be realized through laser, embedded piezoresistive, and pressure sensors (Fig. 7b)^129,142–144^.

Another widely utilized force-sensing structure for thin film tissue is a cantilever beam. By culturing cardiomyocytes on the top surface of a cantilever, the force generated by contraction causes the cantilever to mechanically bend. The deformation of thin films was accurately measured by optical imaging or embedded strain sensors^145^. Flexible cantilever beams were constructed by microfabrication, laser engraving, or 3D printing, in which low elastic modulus materials were used to enhance the deflection^20,146,147^. SU-8 thin-film cantilever beams were easily fabricated via photolithography^148,149^. A laser vibrometer-based measurement system was used to detect the deflection of the cantilever beam driven by single-layer tissue contraction. The resolution ratio of the laser system was ~15 pm^137^. Moreover, the microelectrode could be integrated into the cantilever, allowing real-time mechatronic detection without expensive equipment. The contraction of cardiomyocytes could be converted into a change in the resistance of the strain sensor. The design of embedded sensors in the on-chip sensing system reduced the dependence on external equipment and provided continual in situ monitoring. Parker et al. used a multistep spin-coating process to produce muscle thin films^150,151^. Titanium-gold and carbon black were deposited on the PDMS cantilevers as sensor materials, as shown in Fig. 7c^20,146^. To further improve the sensitivity, a microcrack structure was fabricated by applying a 2% strain to the substrate, which exhibited much higher sensitivity and long-term stability^152^.

#### Thick tissue force sensing

Cells can be encapsulated in hydrogels and self-assemble to form tissue aggregates to construct 3D cardiac tissue with a specific thickness. A certain thickness of cardiac tissue can provide sufficient contraction force to pull a flexible column. For the flexible column model, the contraction force of the tissue can be calculated as follows:

$$\begin{array}{cc}F=\frac{3EI\delta }{{L}^{3}},I=\frac{1}{4}\pi {R}^{4} & {\rm{then}}\,F=\frac{3\pi E{R}^{4}\delta }{4{L}^{3}}\end{array}$$

where the distance moved by one end of the flexible column is set to $\delta$, the length of the flexible column is *L*, the moment of inertia is *I*, and the radius of the column is *R*. Therefore, the contraction force magnitude can be obtained by measuring $\delta$ during the test^153^.

Breakthrough advances in microfabrication have made it possible to engineer 3D thick cardiac tissue models^21,66,125^. The physiological microenvironment of thick cardiac tissue resembles that of native tissue^17,154–158^. The contraction force generated by cardiac tissue is proportional to its cross-sectional area. The tissue contraction can be deduced from the deflection of the beam and the Young’s modulus of its material^21,123^. On the Biowire II platform developed by Radisic, two POMaC wires were fabricated at both ends of the incubation zone, as shown in Fig. 7f^79,159,160^. Tissue contraction values were obtained by measuring the deformation of the flexible wire. Polymer wires maintain a constant Young’s modulus and force–displacement relationship, allowing for accurate force measurements during long-term tissue incubation. A piezoelectric sensitive unit or magnetic components were added to the flexible column to convert deformations into other physical quantities for signal acquisition, as shown in Fig. 7e^98,161,162^.

### Electrophysiological sensors

Electrophysiological sensors monitor changes in electrical potentials during the rhythmic contractions of cardiac tissue. Conventional methods, e.g., voltage clamp, diaphragm clamp, and fluorescence imaging, involve issues concerning invasion and operation difficulty^163–166^. Additionally, methods such as electrochemical impedance spectroscopy or surface plasmon resonance have been utilized for molecular detection^167^; through these methods, biomarkers can be monitored to evaluate tissue status^168,169^. However, these methods are not suitable for continuous high-throughput electrophysiological sensing. Microelectrode arrays (MEAs) are advantageous because extracellular field potential (FP) can be noninvasively measured through an array of microelectrodes in close contact with cells^170–173^. This technology is amenable to on-chip integration and large array fabrication, allowing high-throughput detection of cardiac tissue on a chip^174,175^. Based on the morphology of the electrodes, MEAs are divided into 2D MEAs for single-cell or thin-tissue monitoring and 3D MEAs for thick-tissue monitoring.

The following aspects should be considered when designing MEAs: a. an implement biochemical treatment should be applied on the electrode surface to dictate the interaction between cardiomyocytes and the electrode; b. a meticulous layout should be devised, focusing on two-dimensional sensing and the depth direction; and c. the overall flexibility of the sensor should be ensured to minimize hindrance to tissue contraction. Table 2 summarizes some of the key results of recent research.

**Table 2:** Summary of in vitro MEA applications and example metrics

Material	Technology	Cell type	Number of electrodes	Electrode size	Gap	Impedance @1 kHz	Ref.
Au	Flex-PCB	HL-1	6 × 6	~65 or ~100 μm	380 μm	215.3 ± 70.3 kΩ and 154.7 ± 16.9 kΩ	^195^
Au	Prestressed self-winding technology	Embryonic stem cell-derived CM	-	25 × 25 μm	-	14 ± 7.6 kΩ	^211^
Au	Photolithography, Wet etching	Primary cardiomyocytes from 1-day-old rats	8 × 8	Cap diameter of 1–2 μm	20 μm	-	^187^
Si/Au	Compression Flexion Technique	HL-1	10	-	17.5 μm	-	^210^
Graphene	Photolithography	HL-1	8 × 8	20 μm	1 ± 0.5 × 10^5^ Ω	-	^202^
Carbon nanoparticle ink	Inkjet printing	HL-1	64	30 μm wide	40 μm	10^4^ Ω	^201^

#### 2D MEAs

Noble metals, such as gold and platinum, are generally used to fabricate 2D MEAs that are placed on rigid substrates^176–179^. Wise et al. fabricated the first MEAs in 1970 for biological detection^180^. The device used a silicon wafer as a substrate, and gold was patterned on the substrate by photolithography. The tip diameter of the device was 2 μm, with an electrode spacing of 10–20 μm. The first recording of pulsed electrical signals from isolated neurons promoted the development of MEAs. To improve the sensitivity and reduce the impedance of the metal lead, researchers designed the next generation of microelectrodes by integrating field-effect tube transistors just beneath the microelectrodes.

The most recent research findings on 2D MEAs indicate that extracellular detection devices have rapidly advanced, exhibiting high density, resolution, sensitivity, and enhanced flexibility^181^. The utilization of high-resolution and high-density electrodes is advantageous for the precise examination of electrical signal transmission between tissues. Frey reported a CMOS-based high-density microelectrode array on a chip^182^. The electrode array had a total size of 2.0 × 1.75 mm^2^ and more than 1 × 10^4^ metal electrodes in this area. An average of 3150 electrodes were distributed per square millimeter, and 126 channels could be configured for measurement using a unique switching matrix. This greatly improved the spatial and temporal resolution of MEAs. The voltage sensitivity can be improved by reducing the electrode impedance^183^, which can be achieved by depositing high-specific surface area materials, such as platinum black, nanoporous platinum, and nanoparticles, on the electrodes^184–186^.

**Fig. 8:**
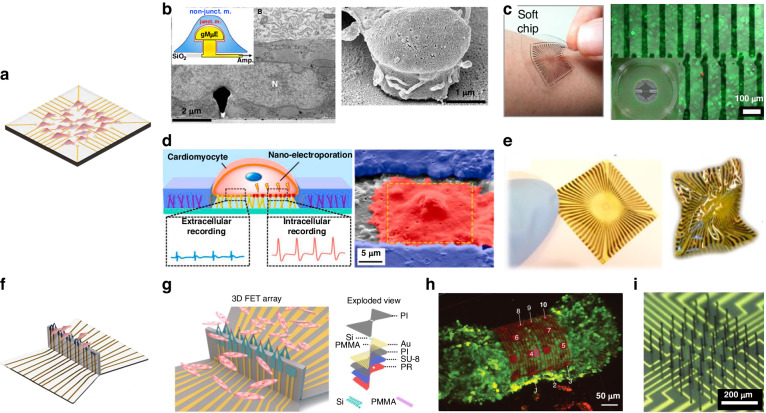
MEA structure diagram. Typical 2D MEA structures: **a** Schematic diagram of 2D MEA; **b** Gold mushroom-shaped microelectrodes^187^; **c** Microelectrode arrays on soft materials^201^; **d** Nanobranched microelectrode array^192^; **e** Flexible Graphene Multielectrode Arrays^202^; 3D MEA structures: **f** Schematic diagram of 3D MEA; **g** 3D transistor arrays^210^; **h** 3D self-rolled biosensor array^211^; **i** Flexible 3D printed microwires^213^

To increase the FP signal amplitude and the sensitivity of MEAs, the sealing resistance between the cell and electrode must be increased. Microelectrodes that invade but do not destroy cells can increase the sealing resistance. Fendyur et al. fabricated MEAs with gold mushroom microelectrodes to increase the sealing resistance between cells and electrodes, as shown in Fig. 8b^16,187–190^. Three photolithographic steps were used to fabricate the wires, mushroom stems, and mushroom caps (electrodes with a height of ~1.5 μm, a cap diameter of 1–2 μm, and a spacing of 20 μm). The mushroom caps of the gold electrodes were functionalized by RGD, readily engulfing the spines; these processes resulted in tight apposition, which enabled “intracellular recording” outside the cell. Apart from altering the electrode structure, MEA-based assisted perforation technology was also employed to capture signals that closely mimic intracellular action potentials^191^. Hu et al. combined the hydrothermal growth method with standard micro- and nanoprocessing to fabricate nanobranched microelectrodes (Fig. 8d), achieving cardiomyocyte electroporation at a harmless low voltage^192^. In comparison to extracellular recordings, the amplitude increased from ~0.4 mV to ~3.0 mV, and the signal-to-noise ratio (SNR) increased ~1.4 times. This form of microelectrode inspired researchers to generate comprehensive and high-quality electrophysiological signals in a minimally invasive manner in subsequent studies^192–194^.

However, rigid metal electrodes or substrates cannot easily form a benign fit between MEAs and cardiac tissue, ultimately leading to a mechanical mismatch^195–200^. This mismatch can affect the shape, organization, and function of cardiomyocytes and lead to inaccurate data from the MEAs^201^. Therefore, flexible materials, such as polyimide (PI) and PDMS, were proposed as substrate materials. Kireev et al. fabricated a versatile flexible graphene electrode array by photolithography (Fig. 8e)^202^. The array used Ti and Au as electrode leads and PI as the substrate and passivation layer material. The flexible MEA provided high-SNR recordings even after severe mechanical deformation. In contrast to PI and other rigid films, stretchable materials, such as PDMS, exhibit superior compliance with soft cells. MEAs based on rigid substrates were compared with those based on PDMS substrates^203^, and the specific surface area of the electrodes increased due to the small bend on the PDMS surface; as a result, the impedance of MEAs on flexible PDMS substrates decreased from 100 Hz to 1 MHz. Adly et al. fabricated flexible MEAs by printing nanoparticle inks on various substrates, including PDMS, agarose, and gelatin, via inkjet printing, as shown in Fig. 8c^201^. Silver nanoparticle ink was used as the electrode feeder and pad, and carbon nanoparticle ink was used to cover the silver feed to form electrodes in contact with the cells. PI was printed on the top as the passivation layer. Extracellular measurements of HL-1 cells with the PDMS-based MEAs revealed signal amplitudes of ~906 μVpp and background noise of ~62 μVpp.

#### 3D MEAs

Compared with 2D cardiac tissue, 3D tissue is more valuable for determining drug response states and capturing signaling pathways in disease states^204–207^. We define 3D MEAs as devices engineered to measure FP signals in the spatial dimensions of thick tissues. In contrast to 2D MEAs, 3D MEAs must be thicker than a single layer of tissue to effectively capture signals in three-dimensional space.

The challenge in realizing 3D microelectrodes lies in the manufacturing of nonplanar microscale structures. The assembly of 3D electrodes was accomplished through mechanically guided substrate deformation, offering a viable approach for recording potential signals in the 3D cellular field^208,209^. A 3D FET array of origami structures was constructed via a compression-folding method, as shown in Fig. 8f^210^. Multilayer 2D precursors were fabricated by standard micro/nanofabrication techniques and bonded onto prestrained elastic substrates. When the restraint of the elastomer was released, the flexure hinge position bent to form the 3D structure. Using this method, a stretchable 128-FET array was fabricated at three different heights. The array was used to measure cellular tissue at three different depths. Similarly, by employing substrate deformation techniques, Kalmykov et al. fabricated 3D self-rolled biosensor arrays (3D-SR-BAs)^211^. Self-rolling polymer structures were used to wrap stem cell-derived engineered heart spheres to study the propagation of electrical signals through the cell spheres, as shown in Fig. 8g. In this work, 3D-SR-BAs were fabricated on silicon wafer planes using micromachining techniques. The shape transition of 3D-SR-BAs was driven by residual mismatch stresses between the different layers. The higher residual stress in the top Cr layer promoted self-coiling of the electrode array during the dissolution of the sacrificial layer. 3D-SR-BAs were used to record CM spheres in 3D with high spatial and temporal resolution. Finally, 3D isochronous maps of the surface of CM spheres were also constructed. The 3D-SR-BAs provided a new idea for the development of 3D microelectrode arrays by substrate deformation.

Another important technology for 3D MEA fabrication is 3D printing^212^. Wu et al. fabricated a microelectrode array with a height of 250 μm and a diameter of 5 μm through directly writing a conductive polymer (Fig. 8h)^213^. The high aspect ratio and tailored elastic modulus allowed the electrodes to be seamlessly embedded into the tissue without impeding pulsation. The incorporation of 3D microelectrodes markedly decreased the impedance and increased the electrochemical activity compared to those of 2D electrodes.

## Conclusions and perspectives

In conclusion, we analyzed the composition of HoCs and provided an overview of the architectural design and advanced manufacturing methods for the typical “3S” components within HoCs. These cardiac tissue scaffolds, stimulation, and sensors can cultivate cardiac tissue with in vivo characteristics and enabling real-time, high-throughput acquisition of various tissue features on the chip. This field is well beyond the proof-of-concept stage. However, HoCs have not been integrated into the current drug development pipeline. Novel technology that involves the development of HoCs has begun to transition from academia to real applications, and these tools have been applied in drug development and cardiac disease modeling^214^. Commercial chips, which exhibit practicality and operability, face limitations due to their low integration, which restricts their large-scale application. At present, HoCs exhibit responses that approximate realistic physiological hallmarks to several standard medications^214,215^. The application and commercialization of HoCs face major challenges, including the following uncertainties: a) how to culture mature cardiac tissues; b) how to track real-time changes in tissue attributes; and c) how to reduce the cost and time necessary to enable high-throughput drug testing. Overcoming these challenges is crucial for the application of HoCs as pivotal processes and alternatives to preclinical animal experiments, which would accelerate drug development.

To increase the maturity of cardiac tissue, a bionic 2D/3D scaffold and an appropriate electrical/mechanical microenvironment are essential components of a chip^216^. The impact of introducing heterogenous elements on the cardiac tissue environment must be carefully considered regardless if scaffolds, stimulation, or sensing components are integrated into chips. Scaffolds and sensors that contact cardiac tissues can constrain tissue beating; thus, materials and structures that match the modulus of cardiac tissues should be favored. The integration of ECM-like scaffolds^217,218^, patterned stimulation^219,220^ and flexible bioelectromechanical sensors^210,221^ on chips inevitably leads to an increase in structural complexity and manufacturing challenges. Nevertheless, as material science progresses and additive manufacturing technology advances, the integration of “3 S” components on a chip offers a promising strategy in the near future. In addition, the development of iPSC and organoid technologies provides referenceable and effective solutions to the limited sources of cardiomyocytes^155,222–224^. With the support of automated microfluidic systems and refined tissue engineering techniques, these an individual customized heart laboratory should provide transformative value to personalized medication and drug development.
